# Patient and Caregiver Experience with Outpatient Palliative Care Telemedicine Visits

**DOI:** 10.1089/pmr.2020.0075

**Published:** 2020-12-28

**Authors:** Brook Calton, William Patrick Shibley, Eve Cohen, Steven Z. Pantilat, Michael W. Rabow, David L. O'Riordan, Kara E. Bischoff

**Affiliations:** ^1^ Division of Palliative Medicine, Department of Medicine, University of California, San Francisco, San Francisco, California, USA.; ^2^ School of Medicine, University of California, San Francisco, San Francisco, California, USA.

**Keywords:** outpatient palliative care, patient satisfaction, telehealth, telemedicine

## Abstract

***Background:*** Telemedicine visits reduce the physical and financial burdens associated with in-person appointments, especially for patients with serious illness. Little is known about patient and caregiver preferences regarding telemedicine visit timing and the discussion of sensitive topics by telemedicine.

***Objective:*** To characterize the experience of patients with serious illness and their caregivers receiving palliative care (PC) by telemedicine.

***Design:*** Mixed-methods telephone survey.

***Setting/Subjects:*** Patients and family caregivers who had at least one telemedicine visit with the outpatient PC team at our urban academic medical center.

***Results:*** A total of 35 patients and 15 caregivers were surveyed. Patient mean age was 61 years, 49% had cancer, and 86% were Caucasian. Caregiver mean age was 62 years. Mean satisfaction with PC telemedicine visits was 8.9 out of 10 for patients; 8.8 for caregivers. Patients (97%) and caregivers (100%) felt comfortable discussing sensitive topics over video. Participants felt telemedicine was an acceptable format to discuss most sensitive topics but 53% of caregivers preferred to receive bad news in person. Participants valued the convenience of telemedicine; they had concerns about rapport building and desired a more user-friendly telemedicine platform.

***Conclusions:*** Patients with serious illness and their caregivers rated telemedicine visits highly and felt comfortable discussing sensitive topics by video. Concerns included rapport building and telemedicine platform setup and quality. The rapid growth of telemedicine during coronavirus disease 2019 creates an imperative for research to understand the impact on the quality of care and mitigate any negative effects of telemedicine within a diverse population of patients.

## Introduction

During coronavirus disease 2019 (COVID-19), telemedicine (real-time videoconferencing between clinicians and patients) has skyrocketed across the United States as a method of providing medical care while limiting virus exposure.^1^ Even before COVID-19, telemedicine was an attractive technology in the palliative care (PC) community. For seriously ill patients with functional, time, and/or financial limitations and those who lack local PC services, telemedicine may increase PC access and reduce the burden of traveling to PC appointments.^2,3^ A growing body of literature suggests PC video consultation is associated with high patient satisfaction, improved symptom burden, and in some cases, lower health care utilization.^4,5^ However, even as telemedicine has become increasingly important in the provision of health care, our understanding of its ideal use and limitations is incomplete.^2,6,7^

In 2014, our quaternary urban academic medical center established a “Telehealth Resource Center” and began providing work relative value units (wRVU) (clinician productivity credits) for all telemedicine encounters regardless of insurance reimbursement to support telemedicine expansion. Our outpatient PC program began offering telemedicine visits in 2016. In 2019, we completed 4840 outpatient PC visits, half of which occurred by telemedicine. Informal feedback on telemedicine in our practice has been overwhelmingly positive. To improve the quality of our telemedicine offerings, we aimed to survey patients and caregivers to characterize their experience with PC telemedicine visits, understand their preferences regarding the timing of telemedicine visits, and their preferred settings to discuss sensitive topics (e.g., discussing difficult news, prognosis, and what to expect near the end of life). At the time of our survey, there were very limited data on these issues.^8,9^ Since our survey's data collection period, understanding these preferences has become even more important because of the rapid growth of telemedicine during the COVID-19 pandemic. Currently, both PC and non-PC clinicians alike are meeting patients for the first time by video and are needing to have sensitive conversations by telemedicine. We hypothesized satisfaction with telemedicine visits as well as preferences regarding the timing and content of telemedicine visits would vary by age—with older patients responding less favorably to telemedicine—and by distance from our medical center—with those living farther away more strongly preferring telemedicine. Differences in telemedicine acceptability and satisfaction by distance from the medical center has been shown in several studies.^10,11^

## Methods

### Subjects

Patients were eligible for the survey if they spoke English and completed at least one visit by telemedicine with one of the two outpatient PC practices (cancer and noncancer). For our cancer PC clinic, we included patients seen from June 15 to 30, 2019 (Refs.^12,13^). To ensure a similar number of surveyed patients from our smaller noncancer clinic, we included patients seen by telemedicine from January to June 2019. We set a goal of surveying 35 patients as we felt this number would be sufficient to identify clinically significant trends and would be a feasible number of interviews for our summer research assistant. Potential caregiver participants were recommended by patients who had completed the survey and by the treating PC team. A trained research assistant used a script to recruit patients and conduct the phone survey. Participation was voluntary and without compensation.

### Data collection

We developed a multiple-item telephone survey synthesizing validated telehealth satisfaction surveys^14–16^ with the National Quality Forum's Palliative Care and End-of-Life Domains.^17^ The survey included questions requiring either quantitative or qualitative responses and was peer-reviewed by our investigator team. Preliminary versions of the survey were administered to three volunteer patients and two volunteer caregivers to assess survey feasibility and obtain feedback that was incorporated into the survey's final version. The final survey contained 22 items for patients and 23 items for caregivers (see Supplementary Appendix SA1 for survey instrument). The UCSF Institutional Review Board approved the project (No. 19-28351); all survey participants provided verbal consent.

Demographic data on patient age, gender, driving distance from their residence to our academic medical center, primary diagnosis, and number of in-person and telemedicine visits with PC were obtained through review of the electronic health record. Caregiver age and gender were self-reported during the survey.

### Analysis

Descriptive statistics, including frequencies, means, and standard deviations, were used to examine the distribution of measures. Chi-square analysis was undertaken to examine bivariate associations between categorical variables and analysis of variance was undertaken to examine associations between categorical and continuous variables. Subgroup analyses by age (≥65 vs. <65 years, driving distance ≥80 km vs. <50 km, cancer vs. noncancer diagnosis, and whether a patient had their first PC visit in person) were conducted with patient data only. The Statistical Package for the Social Sciences version 26 for Mac was used to conduct all analyses.

For the three open-ended questions, thematic analysis based on the framework by Boyatzis^18^ was performed to identify themes and subthemes. After an initial meeting to discuss organization of the thematic analysis, two investigators from our team (W.P.S. and B.C.) individually coded all of the comments for each free-response question, creating their own themes and subthemes. The analysis was considered complete when there was redundancy and saturation of theme identification. The investigators met to discuss the themes and subthemes they each identified and reach agreement on these categories; a third investigator (K.E.B.) mediated any differences of opinion. The investigators categorized the data based on the agreed upon themes and subthemes and met a final time to obtain inter-rater agreement. Frequencies of themes and subthemes were calculated.

## Results

### Participant characteristics

A total of 126 patients (50 patients with cancer and 76 patients without cancer) were initially identified using the data ranges described. Thirty-four patients were excluded by their treating PC team because they were emotionally distressed, dying, or deemed to have communication difficulties. Our telephone-based survey was conducted from July to August 2019. Sixty-five patients were contacted, 25 patients were not reached after three attempts, 5 declined participation, and 35 were surveyed. Survey recruitment stopped after our target of 35 patients was reached (therefore, 27 eligible patients were never contacted). Twenty-two caregivers were contacted, 5 could not be reached after three attempts, 2 declined participation, and 15 were surveyed. The overall response rate among patients and caregivers was 57.5%. There were no significant differences between survey participants and nonparticipants by age (*p* = 0.09), gender (*p* = 0.99), patients' primary diagnosis (*p* = 0.06), or race (*p* = 0.21), or ethnicity (*p* = 0.23). There were no significant differences between patients who participated and those who did not in terms of number of office visits (*p* = 0.11) or the number of video visits they received (*p* = 0.9).

Patient mean age was 61 years, half had cancer (49%), 43% were women, and most were Caucasian (86%) (Table 1). Forty-nine percent of patients had an in-person visit before a telemedicine visit. No patients had an in-person visit after a telemedicine visit. At the time they completed the survey, patients had completed an average of 4.3 telemedicine visits (range 1–21). Caregiver mean age was 62 years, 80% were women, one-quarter (27%) were caring for a patient with cancer.

**Table 1.** Telemedicine Survey Participant Characteristics

Characteristic	Patients (N = 35)	Caregivers (N = 15)
Age, mean (range)	61 (27–83)	62 (45–84)^a^
Female, *n* (%)	15 (43)	12 (80)
Race, *n* (%)		
White or Caucasian	30 (86)	—
Black or African American	3 (9)	—
Asian	1 (3)	—
Other	1 (3)	—
Ethnicity, *n* (%)		
Hispanic or Latino	1 (3)	—
Not Hispanic or Latino	34 (97)	—
Diagnosis, *n* (%)^b,c^		
Cancer	17 (49)	4 (27)
Pulmonary	8 (23)	2 (13)
Liver	6 (17)	3 (20)
Neurologic	4 (9)	5 (33)
Cardiovascular	0	1 (7)
Immunologic	1 (3)	0
Ever received medical care by telemedicine before PC telemedicine visit, *n* (%)	5 (33)	1 (7)
Seen in person by PC before telemedicine visit, *n* (%)^d^	17 (49)	7 (47)
Telemedicine visits, mean (range)	4.3 (1–21)	3.2 (1–8)
PC in-person visits, mean (range)	0.9 (0–5)	2 (0–16)

^a^ Data not collected for one caregiver.

^b^ For caregivers, diagnosis is that of the patient they are caring for.

^c^ Percentages may not sum to 100 due to rounding.

^d^ No patients were seen in person who were not seen in person initially.

PC, palliative care.

We compared demographic data for all PC patients who were seen at least once by telemedicine (*n* = 488) with PC patients who only received in-person visits (*n* = 560) between January and June 2019. Patients seen by telemedicine were younger (mean age 61.1 vs. 64.6 years, *p* < 0.0001) and more likely to be white (70.9% vs. 63%, *p* = 0.05) and English speaking (95.1% vs. 89.5%, *p* = 0.001) than patients only seen in person. There were no statistically significant differences by gender (*p* = 0.33) or ethnicity (*p* = 0.94).

### Telemedicine visit satisfaction

Mean telemedicine satisfaction score on a 10-point scale (0 = “not satisfied at all” to 10 = “completely satisfied”) was 8.9 for patients (95% confidence interval [CI]: 8.5–9.3) and 8.8 for caregivers (95% CI: 8.0–9.6). There were no differences in telemedicine visit satisfaction between patients and caregivers (*p* = 0.88) or by patients' age (*p* = 0.07), distance from the medical center (*p* = 0.84), cancer diagnosis (*p* = 0.69), or whether the first visit was in person (*p* = 0.20).

Nearly all participants reported that they would have another PC telemedicine visit if it was offered with no difference between patients (97%, *n* = 34) and caregivers (100%, *n* = 15; *p* = 0.51) (Table 2). There were no significant differences between patients and caregivers in recommending having a PC visit by telemedicine to others (86%, *n* = 30 vs. 93%, *n* = 14; *p* = 0.45). All patients and 93% of caregivers reported that it is easy to communicate with the PC team over telemedicine and 86% (*n* = 30) of patients, and 93% (*n* = 14) of caregivers reported the telemedicine technology was easy to use. There were no significant differences by patient subgroups.

**Table 2.** Telemedicine Satisfaction Comparing Patient (*N* = 35) and Caregiver (*N* = 15) Responses

	Patients, n (%)	Caregivers, n (%)	χ^2^	p^*^
I would do another video visit if it were offered by my palliative care team				
Strongly disagree/disagree/neutral	1 (3)	1 (7)	0.02	0.88
Strongly agree/agree	34 (97)	14 (93)		
I would recommend receiving palliative care by video visit to others				
Strongly disagree/disagree/neutral	5 (14)	1 (7)	0.58	0.45
Strongly agree/agree	30 (86)	14 (93)		
It was easy for me to communicate with my palliative care team during my video visit(s)				
Strongly disagree/disagree/neutral	0	1 (7)	2.38	0.12
Strongly agree/agree	35 (100)	14 (93)		
The video visit technology was easy to use				
Strongly disagree/disagree/neutral	5 (14)	1 (7)	0.58	0.45
Strongly agree/agree	30 (86)	14 (93)		

^*^ For comparison between patient and caregiver responses.

### Telemedicine visit timing

A greater percentage of caregivers (60%, *n* = 9 of 15) than patients (25%, *n* = 9 of 35) agreed the first PC appointment should be in person in clinic (*p* = 0.02). Forty-four percent of patients (*n* = 7 of 16) living closer than 50 miles from the medical center agreed an initial in-person visit was important versus only 11% of patients living farther than 50 miles (*n* = 2 of 19; *p* = 0.03). Furthermore, patients who had their first PC visit in person (*n* = 10, 59%) were more likely to feel an initial in-person visit was important than patients who were seen exclusively by telemedicine (*n* = 7, 41%, *p* = 0.04). Most patients (71%) and caregivers (67%) reported feeling comfortable having all of their PC appointments by telemedicine (*p* = 0.51).

### Sensitive conversations

**Fig. 1.**
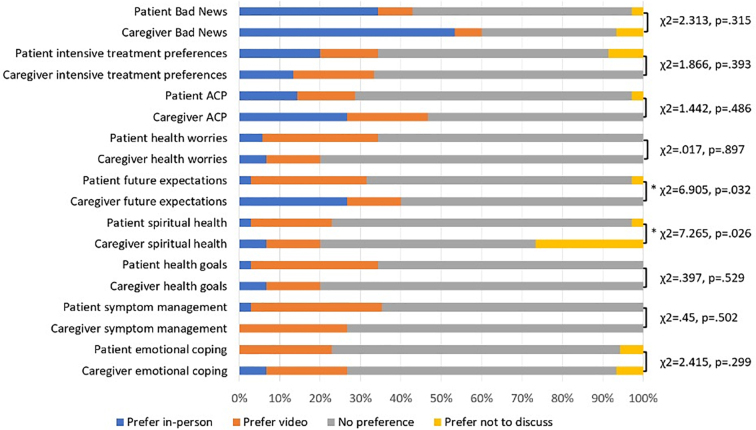
Communication preferences for discussion of sensitive topics. *Denotes statistically significant difference where *p* < 0.05.

Ninety-seven percent of patients and 100% of caregivers felt comfortable discussing sensitive topics by telemedicine (*p* = 0.51). These results were consistent across patient subgroups. Participants felt telemedicine was an acceptable, and often preferable, format to discuss most sensitive topics (Fig. 1). For patients, the only topic for which more than one-quarter of patients said they would want an in-person visit was receiving bad news (34%); for caregivers, the topics were receiving bad news (53%), advance care planning (27%), and what to expect in the future (27%).

### Open-ended responses

There were 116 comments to the open-ended question asking patients and caregivers what they liked about receiving PC by telemedicine (Table 3). The most common themes were convenience (53 comments, 46%), that patients and caregivers felt telemedicine visits were equivalent or better than other forms of communication, including telephone and in-person visits (32 comments, 28%), and enhanced access to care (21 comments, 18%). Regarding convenience, respondents appreciated the time-savings of telemedicine visits (33 comments, 62%) and comfort of doing a visit in one's own home (11 comments, 21%) (with several mentions of being able to attend the visit in pajamas). Describing how telemedicine visits felt equivalent or better to other types of settings, one caregiver said, “Unless there is a compelling reason to be there in person such as physical examinations, or laboratories, a video visit is a preferred way for us to do the interaction, especially when patients are dealing with mobility.”

**Table 3.** Patient and Caregiver Reported Strengths and Downsides of Palliative Care Telemedicine Visits

Theme (N by theme, % of total)^a^	Subtheme (N, % of theme)^a^	Selected patient/caregiver comment
Strengths (116 comments total)		
Convenience (53, 46)	Time-savings (33, 62)	“I like that it's an option because it meant we didn't have to make the 2–3 hour drive to San Francisco. I like that it's out there because we wouldn't be able to do it if they had to go to SF every time.” (Caregiver)
	Personal comfort (11, 21)	“It's more comfortable. You can be in your pajamas with a cup of coffee if you want. If the doctor is running late, I can do other things at my leisure at home.” (Patient)
	General comments re: convenience (5, 9)	“This is very convenient and helpful.” (Caregiver)
	Cost-savings (4, 8)	“I was astonished to find out that [the video visit] was at no cost to us. It saved significant expense because it saved a drive, food, and a hotel for 2 nights.” (Caregiver)
Comparisons with other forms of communication (32, 28)	Comparisons with in-person visits (15, 47)	“Unless there is a compelling reason to be there in person such as physical exams, or labs, a video visit is a preferred way for have the interaction, especially when patients are dealing with mobility.” (Caregiver)
	Unique features of video visits (12, 38)	“Gives me a lot of time to think about how I'm really feeling. I'm more reflective at home. It's easy to go grab my meds at home…” (Patient)
	Comparisons with telephone encounters (5, 16)	“It feels a lot more intimate than a phone call. Seeing people face to face enhances the visit. It's like you are in the room with them.” (Patient)
Enhanced access (21, 18)	For symptomatic or disabled patients (10, 48)	“My husband is in a wheelchair and on a ventilator, video appointments avoid having to transport him for a visit.” (Caregiver)
	Greater frequency of communication with medical team (8, 38)	“It's easier to stay up-to-date.” (Patient)
	To specialty PC services (3, 14)	“I've spent a lot of time trying to track down any local palliative care services and we've found nothing [near home].” (Caregiver)
Technology (10, 9)	Ease of use (6, 60)	“It is easy to use.” (Patient)
	Video platform quality (4, 40)	“You can say and hear everything you need to.” (Patient)
Downsides (76 comments total)		
Technology concerns (26, 34)	Video visit platform (18, 69)	“The technology didn't work and it wasted a lot of time…we had to move to a phone-call.” (Patient)
	Tech-literacy (5, 19)	“Some people might be intimidated about doing it over video…” (Patient)
	Video visit tech setup (3, 12)	“It requires that you have things at home to have a high quality video conference. Need good wifi, a good camera, a good screen.” (Caregiver)
Relationships and rapport (21, 28)	Rapport building (12, 57)	“It's not as intimate as far as communicating 1:1. I don't feel like I get to know the doctor as well compared to in-person appointments.” (Patient)
	Nonverbal communication (4, 19)	“You can see faces but sometimes you miss the body language.” (Patient)
	Value in initial in-person visit (3, 14)	“Technology creates a little distance, but it's not a big deal if you've met the person [in-person first].” (Caregiver)
	Discussing sensitive topics (2, 10)	“With sensitive topics you feel a little less empathy.” (Caregiver)
Limitations in scope of services (15, 20)	N/A	“Losing the proximity with other human beings is a real disadvantage. You can lose the more subtle signals of how people are doing over video.” (Patient)
No downsides! (14, 18)	N/A	“None, I prefer them [video visits].” (Patient)

^a^ Percentages may not sum to 100 due to rounding.

N/A, not applicable.

There were 76 comments about perceived downsides of receiving PC by telemedicine (Table 3). A key theme that emerged was technology (26 comments, 34%). Another theme that emerged was concern about relationship and rapport building by telemedicine (21 comments, 28%). Three respondents noted limited opportunities to see body language by video.

There were 60 comments by patients and caregivers on areas for improvement with PC telemedicine visits (Table 4). Notably 19 patients and caregivers reported they could not think of a way to improve PC telemedicine visits. Half of the comments focused on technological improvements with participants desiring more help setting up the technology (30 comments, 50%), an improved telemedicine platform quality (11 comments, 37%), and real-time technology support (4 comments, 13%).

**Table 4.** Patient and Caregiver Reported Areas for Improvement of Telemedicine

Theme (N by theme, % of total)^a^	Subtheme (N, % of theme)^a^	Example comment
Ideas to improve PC telemedicine (60 comments total)		
Technology (30, 50)	Video visit tech setup (15, 50)	“The technology might be hard for some people. Making the system as foolproof as possible would be a good idea. It was easy for me, but I can see how it could be frustrating for people with less tech experience.” (Patient)
	Video visit platform quality (11, 37)	“It can be hard for older people to do this with technology. It would be great to simplify the experience for older adults.” (Patient)
	Real-time tech support (4, 13)	“Have an IT resource who can help patients and families who are having trouble, especially if people aren't as familiar with video conferencing.” (Caregiver)
No ideas! (19, 32)	N/A	“Nothing I can think of. It's basic but it's effective. If it ain't broke don't fix it.” (Patient)
Miscellaneous (6, 10)	N/A	“Make sure [the team] has a sense of the local resources available and the local health care environment for people who don't live near SF.” (Patient)
Relationships and rapport (5, 8)	N/A	“The visibility of seeing all three of the team, but even when one of the team members is asking the questions, the camera should be on them just for eye contact.” (Patient)

^a^ Percentages may not sum to 100 due to rounding.

IT, information technology.

## Discussion

The experience of surveyed patients with serious illness and family caregivers seen by PC telemedicine at our urban academic medical center was overwhelmingly positive. Patient and caregiver satisfaction was high. The majority of patients and caregivers would do another telemedicine visit if offered and would recommend receiving PC by telemedicine to others. Consistent with past research, convenience^5,9,19^ (both time and cost-savings) and enhanced access to the medical team^10,11^ (especially for those with functional limitations or who lacked specialty PC near their home) were reported as key advantages of telemedicine in our survey. Participants also frequently noted telemedicine visits felt equivalent or better than in-person visits.

Survey respondents offered a range of perspectives on the effectiveness of rapport building by telemedicine. One-third of the participants' comments on telemedicine limitations acknowledged concerns about rapport building by video. One patient remarked telemedicine visits are, “still a cold experience. It is not a warm handshake. It is not human to human contact.” Another patient offered an alternative perspective, “It's almost like being with the person since you can see the person on the other side and we can talk freely.” To date, the impact of telemedicine on patient–family–clinician rapport has not been adequately characterized in the literature.^19^

Although some clinicians assert an initial in-person visit is necessary to establish rapport,^8^ only one-quarter of surveyed patients felt this was important. Patients living closer to the medical center or who had their first PC visit in person were significantly more likely to feel an initial in-person visit was important. Notably, the telemedicine satisfaction scores for patients seen exclusively by telemedicine were as high as those seen by the PC team in person initially. Caregivers were more likely than patients to feel an in-person visit was important. Caregivers may feel a full assessment in person is needed to provide comprehensive care or patients who are symptomatic find the burden of travelling to clinic outweigh any potential advantages of in-person visits.

As our health care system increasingly relies on telemedicine during and after COVID-19, research to understand the impact of telemedicine and the timing of telemedicine visits on patient–clinician rapport and key health care outcomes is needed. In the meantime, universal clinician training on telemedicine best practices such as ensuring good lighting and orienting the patient to the clinician's location^2^ support rapport building by video. Ensuring telemedicine technology works smoothly (a limitation and area for improvement often cited by our survey participants) can also promote rapport building by avoiding technology-related frustration and supporting the clinician in effectively identifying patient and caregiver verbal and nonverbal cues.

The most striking finding from our survey was how comfortable patients and caregivers felt discussing sensitive or emotional topics by video (97% of patients and 100% of caregivers reported feeling comfortable). With few exceptions, survey participants did not prefer in-person visits for the discussion of nine sensitive topics. The only topic for which more than one-quarter of patients wanted an in-person visit was receiving bad news (34%). For caregivers, the topics that more than one-quarter preferred discussing in person were bad news (53%), advance care planning (27%), and what to expect in the future (27%). Further study on this topic is warranted. It is unclear whether patients and caregivers are truly as comfortable discussing sensitive topics through telemedicine, or whether the convenience and timeliness of telemedicine outweigh comfort gained from an in-person visit. In a study of telemedicine at an academic medical center, some primary care patients reported a preference to receive difficult news by video because they felt they would receive the news earlier than coming to clinic and/or their home provided more comfort, social support, or privacy.^9^

Our medical center's experience and anecdotal reports from PC and non-PC teams across the country suggest more difficult conversations are being held by telemedicine during COVID-19.^6,20^ Additional data to understand the quality of sensitive conversations held by telemedicine and how they may differ from in-person conversations are needed to guide telemedicine best practices and clinician trainings.^21^ Future research should also investigate whether patient outcomes such as pain management and completion of advance care planning documents are different for patients cared for by telemedicine versus in person. As we await these data, general and COVID-specific communication frameworks offered by VitalTalk^22^ and Ariadne Lab's Serious Illness Conversation Project^23^ can support clinicians as they have serious illness conversations in person and by telemedicine.

### Strengths and limitations

Our survey is the first to provide a detailed report on patient and caregiver perspectives on the timing of telemedicine visits and preferences for communication about sensitive topics by video. We present quantitative and qualitative perspectives to offer a fuller picture of their experience. Our participants represented a broad range of ages and diagnoses.

Our findings are tempered by the following limitations. We had a small convenience sample size—we did not survey all individuals who completed a telemedicine visit during our study's time period. Our participants were predominantly white/Caucasian and English-speaking, limiting generalizability to other populations. Until very recently, we were unable to include interpreters in our telemedicine visits and this in part explains our survey population's homogeneity. Our survey occurred before COVID-19. The demographics and perspectives of patients completing telemedicine visits before versus during the pandemic may differ. We did not survey a sizable number of patients who may have completed telemedicine visits but for whom their treating clinician asked us not to survey because they were emotionally distressed or dying, which may have led to selection bias. Patients were not randomized to receive PC by telemedicine, which may make our results look more positive than with a less eager or technologically savvy group of patients and caregivers. To this point, it is notable that our patients who completed at least one telemedicine visit were more likely to be white, younger, and speak English than our patients seen exclusively in person. Deepening our understanding of the perspectives of patients from diverse groups on telemedicine and developing creative strategies to address barriers that exist is critical to prevent disparities in access to care.

## Conclusions

Telemedicine visits were highly rated by our outpatient PC patients and caregivers. Participants appreciated the convenience telemedicine visits offer and frequently cited telemedicine visits felt equivalent to other forms of communication, including in-person visits. Patients and caregivers felt comfortable discussing a wide range of sensitive topics by telemedicine. Attention to telemedicine platform quality and technology setup is needed to optimize clinician–patient communication and rapport building. To ensure patients and families receive the best care possible, research within a large population of patients from a diversity of backgrounds is needed to understand the impact of telemedicine on rapport, patient care, and health care outcomes. This information can guide telemedicine quality improvement efforts, the development of best practices, and clinician trainings.

## Supplementary Material

Supplemental data

## Supplementary Material

Supplementary Appendix SA1

## Abbreviations Used

ACP: advance care planning
CI: confidence interval
COVID-19: coronavirus disease 2019
PC: palliative care

## References

[1] Webster P: Virtual health care in the era of COVID-19. Lancet Lond Engl 2020;395:1180–1181 10.1016/S0140-6736(20)30818-7 PMC7146660 32278374

[2] Calton BA, Rabow MW, Branagan L, et al.: Top ten tips palliative care clinicians should know about telepalliative care. J Palliat Med 2019;22:981–985 31237467 10.1089/jpm.2019.0278

[3] Bonsignore L, Bloom N, Steinhauser K, et al.: Evaluating the feasibility and acceptability of a telehealth program in a rural palliative care population: TapCloud for palliative care. J Pain Symptom Manage 2018;56:7–14 29551433 10.1016/j.jpainsymman.2018.03.013

[4] Worster B, Swartz K: Telemedicine and palliative care: An increasing role in supportive oncology. Curr Oncol Rep 2017;19:37 28417310 10.1007/s11912-017-0600-y

[5] Tasneem S, Kim A, Bagheri A, Lebret J: Telemedicine video visits for patients receiving palliative care: A qualitative study. Am J Hosp Palliat Care 2019;36:789–794 31064195 10.1177/1049909119846843

[6] Wolf I, Waissengrin B, Pelles S: Breaking bad news via telemedicine: A new challenge at times of an epidemic. Oncologist 2020;25:e879–e880 32304624 10.1634/theoncologist.2020-0284 PMC7288637

[7] Bashshur R, Doarn CR, Frenk JM, et al.: Telemedicine and the COVID-19 pandemic, lessons for the future. Telemed J E-Health Off J Am Telemed Assoc 2020;26:571–573 10.1089/tmj.2020.29040.rb 32275485

[8] Cowan KE, McKean AJ, Gentry MT, Hilty DM: Barriers to use of telepsychiatry: Clinicians as gatekeepers. Mayo Clin Proc 2019;94:2510–2523 31806104 10.1016/j.mayocp.2019.04.018

[9] Powell RE, Henstenburg JM, Cooper G, et al.: Patient perceptions of telehealth primary care video visits. Ann Fam Med 2017;15:225–229 28483887 10.1370/afm.2095 PMC5422083

[10] Wood PR, Caplan L: Outcomes, satisfaction, and costs of a rheumatology telemedicine program: A longitudinal evaluation. J Clin Rheumatol Pract Rep Rheum Musculoskelet Dis 2019;25:41–44 10.1097/RHU.0000000000000778 30461466

[11] Gardner MR, Jenkins SM, O'Neil DA, et al.: Perceptions of video-based appointments from the patient's home: A patient survey. Telemed J E-Health Off J Am Telemed Assoc 2015;21:281–285 10.1089/tmj.2014.0037 PMC4378342 25166260

[12] Bischoff K, Yang E, Kojimoto G, et al.: What we do: Key activities of an outpatient palliative care team at an academic cancer center. J Palliat Med 2018;21:999–1004 29431580 10.1089/jpm.2017.0441

[13] Bischoff K, Weinberg V, Rabow MW: Palliative and oncologic co-management: Symptom management for outpatients with cancer. Support Care Cancer Off J Multinatl Assoc Support Care Cancer 2013;21:3031–3037 10.1007/s00520-013-1838-z 23794100

[14] University of Washington: UW Telemedicine Satisfaction Survey. HealthOnline. https://healthonline.washington.edu/sites/default/files/record_pdfs/UW-Telemedicine-Patient-Satisfaction-Survey.pdf (Last accessed 111, 2020)

[15] Bakken S, Grullon-Figueroa L, Izquierdo R, et al.: Development, validation, and use of English and Spanish versions of the telemedicine satisfaction and usefulness questionnaire. J Am Med Inform Assoc 2006;13:660–667 16929036 10.1197/jamia.M2146 PMC1656962

[16] Yip MP, Chang AM, Chan J, MacKenzie AE: Development of the Telemedicine Satisfaction Questionnaire to evaluate patient satisfaction with telemedicine: A preliminary study. J Telemed Telecare 2003;9:46–50 12641893 10.1258/135763303321159693

[17] Ferrell BR, Twaddle ML, Melnick A, Meier DE: National consensus project clinical practice guidelines for quality palliative care guidelines. J Palliat Med 2018;21:1684–1689 30179523 10.1089/jpm.2018.0431

[18] Boyatzis RE: Transforming Qualitative Information: Thematic Analysis and Code Development. Thousand Oaks, CA: Sage Publications, 1998

[19] Donelan K, Barreto EA, Sossong S, et al.: Patient and clinician experiences with telehealth for patient follow-up care. Am J Manag Care 2019;25:40–44 30667610

[20] Flint L, Kotwal A: The new normal: Key considerations for effective serious illness communication over video or telephone during the coronavirus disease 2019 (COVID-19) pandemic. Ann Intern Med 2020;173:486–488 32422084 10.7326/M20-1982 PMC7236893

[21] Calton B, Abedini N, Fratkin M: Telemedicine in the time of coronavirus. J Pain Symptom Manage 2020;60:e12–e14 10.1016/j.jpainsymman.2020.03.019 PMC7271287 32240756

[22] VitalTalk: VitalTalk Resources. https://www.vitaltalk.org/resources (Last accessed 111, 2020)

[23] Ariadne Labs: Serious Illness Conversation Guide. https://www.ariadnelabs.org/wp-content/uploads/sites/2/2017/05/SI-CG-2017-04-21_FINAL.pdf (Last accessed 111, 2020)

