# Supplementary material for: Patient and Caregiver Experience with Outpatient Palliative Care Telemedicine Visits
Source: Palliat Med Rep. 2020 Dec 28;1(1):339–46. doi: 10.1089/pmr.2020.0075 (PMC8241370; doi:10.1089/pmr.2020.0075)
Supplement: Supplemental data [file Supp_AppA1.pdf]

Appendix 1. Telemedicine Telephone Survey

Default Question Block

Survey start time:

Before we get started, we are doing a similar survey with caregivers. Did you have any caregivers, family members, or loved one's participate in video appointments with you?

Yes

No

Do you think they would be willing to participate in a survey like this?

Yes

Maybe

No

Would you be willing to share their name and phone number with us?

Was(were) your video visit(s) with the *symptom management service* the first time you ever received medical care by video visit:

Yes

No

If answer is NO, what other clinicians did you see over video and approximately how many times?

Prior to your first video visit with the symptom management service the primary emotion you were feeling about having a video visit was:

Prior to [INSERT PATIENT NAME]'s first video visit with the symptom management service the primary emotion you were feeling about having a video visit was:

Unsure

Nervous

Excited

Curious

Indifferent

Rate the extent to which you agree with the following statemnt (strongly disagree, disagree, neutral, agree, strongly agree):

	Strongly Disagree	Disagree	Neutral	Agree	Strongly Agree
It was easy for me to communicate with my symptom management team during my video visit(s). |||||||||||||||||||||||||| It was easy for me to communicate with the symptom management team during the video visit(s).	O	O	O	O	O

	Strongly Disagree	Disagree	Neutral	Agree	Strongly Agree
The video visit technology was easy to use.	O	O	O	O	O
I feel comfortable discussing sensitive or emotional topics with my symptom management team by video.	O	O	O	O	O
I think the first visit with the symptom management team should always be in-person in clinic (i.e. not by video)	O	O	O	O	O
I would be open to having all of my symptom management appointments by video (i.e. never coming to clinic for these appointments). |||||||||||||||||||||||||| I would be open to having all of [INSERT PATIENT NAME]'s symptom management appointments by video (i.e. never coming to clinic for these appointments).	O	O	O	O	O
I would do another video visit if it were offered by my symptom management team.	O	O	O	O	O
I would recommend receiving symptom management by video visit to others.	O	O	O	O	O

On a scale of 1-10 with 1 being not satisfied at all and 10 being completely satisfied, what was your overall satisfaction with *your symptom management* video visits (1-10)

1

2

3

4

5

6

7

8

9

10

As a patient, what do you like about receiving *symptom management* by video visit: (what else until exhausted)

As a caregiver, what do you like about receiving symptom management by video visit: (what else until exhausted)

As a patient, what are the downsides of receiving *symptom management* by video visit : (what else until exhausted)

As a caregiver, what are the downsides of receiving *symptom management* by video visit include: (what else until exhausted)

I'm going to list a number of topics you might discuss with your *symptom management* team. Please let me know if you'd prefer to discuss each topic at an in-person clinic appointment, by video visit, either in-person or over video is OK, or you would prefer not to discuss the topic at all regardless of format.

<table>
<tr><th></th><th>Prefer to discuss in-person</th><th>Prefer to discuss over video</th><th>Either in-person or video are OK</th><th>Would prefer not to discuss at all</th></tr>
<tr><td>What to expect in the future: |||||||||||||||||||||| What to expect in the future [INSERT PATIENT NAME]'s health</td><td>O</td><td>O</td><td>O</td><td>O</td></tr>
<tr><td>Your goals as they pertain to your health |||||||||||||||||||||||||||||| Your goals as they pertain to [INSERT PATIENT NAME]'s health</td><td>O</td><td>O</td><td>O</td><td>O</td></tr>
<tr><td>Your worries about your health ||||||||||||||||||||||| Your worries about [INSERT PATIENT NAME]'s health</td><td>O</td><td>O</td><td>O</td><td>O</td></tr>
<tr><td>Symptom managment</td><td>O</td><td>O</td><td>O</td><td>O</td></tr>
<tr><td>Your preferences for intensive medical treatments including resuscitation (aka CPR) and being put on a breathing machine ||||||||||||||||||||| [INSERT PATIENT NAME]'s preferences for intensive medical treatments including resuscitation (aka CPR) and being put on a breathing machine</td><td>O</td><td>O</td><td>O</td><td>O</td></tr>
<tr><td>Completion of an advance care planning document such as an advance directive or POLST (pink form, Physician's Orders for Life-Sustaining Treatment)</td><td>O</td><td>O</td><td>O</td><td>O</td></tr>
</table>

<table>
<tr><th></th><th>Prefer to discuss in-person</th><th>Prefer to discuss over video</th><th>Either in-person or video are OK</th><th>Would prefer not to discuss at all</th></tr>
<tr><td>Receiving bad news about your health |||||||||||||||||||||||||| Receiving bad news about [INSERT PATIENT NAME]'s health</td><td></td><td></td><td></td><td></td></tr>
<tr><td>How you're coping emotionally with your health ||||||||||||||||||||||||||||||||||| How you're coping emotionally with [INSERT PATIENT NAME]'s health</td><td></td><td></td><td></td><td></td></tr>
<tr><td>Your spiritual health and/or religious practices</td><td></td><td></td><td></td><td></td></tr>
<tr><td>[CAREGIVER ONLY] Receiving support from the symptom management team regarding your role as a caregiver</td><td></td><td></td><td></td><td></td></tr>
</table>

What ideas do you have about ways to improve symptom management video visits for patients and families?

End time:

Other comments about interview:

This is the SMS Patient Telehealth Survey

Date:

Name of patient/caregiver:

Age:

Patient or caregiver:

Patient

Caregiver

If caregiver - name of caregiver's relevant patient

Service used:

Palliative Care Service

Symptom Management Service

Gender:

Male

Female

Other/decline to state

Patient Diagnosis:

Cardiac

Cardiovascular

Cancer

Complex chronic conditions/FTT

Congenital/Chromasomal

Gastrointestinal

Hematology

Hepatic

Pulmonary

Renal

Vascular

Other:

Dates of video visits:

Number of video visits:

Number of office visits:

Patient was seen in-person prior to the first video visit

Yes

No

Powered by Qualtrics
